# Supplementary material for: Hybrid Models and Biological Model Reduction with PyDSTool
Source: PLoS Comput Biol. 2012 Aug 9;8(8):e1002628. doi: 10.1371/journal.pcbi.1002628 (PMC3415397; doi:10.1371/journal.pcbi.1002628)
Supplement: Text S4 — Complete source code for the PyDSTool package (version 0.88.120504). Includes API documentation and help files linking to web pages. This file is identical to the current public release on Sourceforge.net. (ZIP) [file pcbi.1002628.s004.zip › PyDSTool/html/PyDSTool.PyCont.misc-module.html]

xml version="1.0" encoding="ascii"?


PyDSTool.PyCont.misc


| Home | Trees | Indices | Help | PyDSTool |
| --- | --- | --- | --- | --- |

Package PyDSTool ::
Package PyCont ::
Module misc

[hide private]

[frames] | no frames]

# Module misc

source code

Common functions

Drew LaMar, March 2006


|  |  |
| --- | --- |
| Classes | [hide private] |

IterationError

Struct


|  |  |
| --- | --- |
| Functions | [hide private] |

|  |  |
| --- | --- |
| iszero(x, y) | source code |

|  |  |
| --- | --- |
| isnotzero(x, y) | source code |

|  |  |
| --- | --- |
| monotone(x, num=None, direc=1)  Checks to see if the list 'x' is increasing/decreasing ('direc' = 1/-1, respectively). | source code |

|  |  |
| --- | --- |
| todict(C, X) | source code |

|  |  |
| --- | --- |
| tocoords(C, D) | source code |

|  |  |
| --- | --- |
| jac(func, x0, ind)  Compute (n-1) x m Jacobian of function func at x0, where n = len(x0) and m = len(ind). | source code |

|  |  |
| --- | --- |
| hess(func, x0, ind)  Computes second derivative using 2nd order centered finite difference scheme. | source code |

|  |  |
| --- | --- |
| hess3(func, x0, ind)  Computes third derivative using hess function. | source code |

|  |  |
| --- | --- |
| bilinearform(A, x1, x2) | source code |

|  |  |
| --- | --- |
| trilinearform(A, x1, x2, x3) | source code |

|  |  |
| --- | --- |
| ijtoind(i, j)  0 <= j < i | source code |

|  |  |
| --- | --- |
| indtoij(ind) | source code |

|  |  |
| --- | --- |
| testindij(n) | source code |

|  |  |
| --- | --- |
| wedge(u, v) | source code |

|  |  |
| --- | --- |
| invwedge(q, n) | source code |

|  |  |
| --- | --- |
| bialttoeig(q, p, n, A) | source code |

|  |  |
| --- | --- |
| firstlyapunov(X, F, w, J\_coords=None, V=None, W=None, p=None, q=None, check=False) | source code |

|  |  |
| --- | --- |
| CheckHopf(C, X) | source code |

|  |  |
| --- | --- |
| unique(s)  Return a list of the elements in s, but without duplicates. | source code |

|  |  |
| --- | --- |
| partition(a, elems)  Current issue with neutral points changing past bifurcation point. | source code |

|  |  |
| --- | --- |
| negate(x) | source code |

|  |  |
| --- | --- |
| getFlowJac(pt, verbose=False) | source code |

|  |  |
| --- | --- |
| getFlowMaps(n, pt, pttype, method=`'``standard``'`)  method: 'standard' is currently the only working method for calculating the flow maps. | source code |

|  |  |
| --- | --- |
| getLeftEvecs(n, ntst, maps, flow\_vecs, method=`'``standard``'`, verbose=False)  Get left eigenvetors w corresponding to the unit eigenvalue of flow, normalized so that w.v = 1, where v are flow vectors. | source code |

|  |  |
| --- | --- |
| evec1\_standard(idxs, evals, levecs)  Standard method | source code |


|  |  |
| --- | --- |
| Variables | [hide private] |

all\_point\_types = `['P', 'RG', 'LP', 'BP', 'H', 'BT', 'ZH', 'CP...`

all\_curve\_types = `['EP', 'LP', 'H', 'FP', 'LC']`

divide = `<ufunc 'divide'>`

subtract = `<ufunc 'subtract'>`

conjugate = `<ufunc 'conjugate'>`

sqrt = `<ufunc 'sqrt'>`

Inf = `inf`

NaN = `nan`

isfinite = `<ufunc 'isfinite'>`

r\_ = `<numpy.lib.index_tricks.RClass object at 0x11542f0>`

c\_ = `<numpy.lib.index_tricks.CClass object at 0x1154330>`

sign = `<ufunc 'sign'>`

mod = `<ufunc 'remainder'>`

multiply = `<ufunc 'multiply'>`

PI = `3.14159265359`

\_classes = `['IterationError', 'Struct']`

\_functions = `['iszero', 'isnotzero', 'todict', 'tocoords', 'ja...`

FLOQ\_TOL = `0.01`


|  |  |
| --- | --- |
| Function Details | [hide private] |

|  |  |
| --- | --- |
| monotone(x, num=None, direc=1) | source code |

Checks to see if the list 'x' is increasing/decreasing ('direc' =
1/-1, respectively). If 'num' is specified, then it checks the first
'num' indices in 'x' if num > 0 and the last 'num' indices in 'x' if
num < 0.

|  |  |
| --- | --- |
| jac(func, x0, ind) | source code |

Compute (n-1) x m Jacobian of function func at x0, where n = len(x0)
and m = len(ind). ind denotes the indices along which to compute the
Jacobian.

NOTE: This assumes func has 1 more variable than it has
equations!!

|  |  |
| --- | --- |
| unique(s) | source code |

Return a list of the elements in s, but without duplicates.

For example, unique([1,2,3,1,2,3]) is some permutation of [1,2,3],
unique("abcabc") some permutation of ["a",
"b", "c"], and unique(([1, 2], [2, 3], [1, 2])) some
permutation of [[2, 3], [1, 2]].

For best speed, all sequence elements should be hashable. Then
unique() will usually work in linear time.

If not possible, the sequence elements should enjoy a total ordering,
and if list(s).sort() doesn't raise TypeError it's assumed that they do
enjoy a total ordering. Then unique() will usually work in O(N\*log2(N))
time.

If that's not possible either, the sequence elements must support
equality-testing. Then unique() will usually work in quadratic time.

Author: Tim Peters (2001/04/06)

|  |  |
| --- | --- |
| partition(a, elems) | source code |

Current issue with neutral points changing past bifurcation point.
False advertising as well: Not really a general function (specialized for
my use)

|  |  |
| --- | --- |
| getLeftEvecs(n, ntst, maps, flow\_vecs, method=`'``standard``'`, verbose=False) | source code |

```
Get left eigenvetors w corresponding to the unit eigenvalue of flow,
normalized so that w.v = 1, where v are flow vectors.

method: 'standard' is currently the only working method for calculating
  the flow maps and left eigenvectors.
```

  


|  |  |
| --- | --- |
| Variables Details | [hide private] |

### all\_point\_types

Value:
:   ```
    ['P',
     'RG',
     'LP',
     'BP',
     'H',
     'BT',
     'ZH',
     'CP',
    ...
    ```

### \_functions

Value:
:   ```
    ['iszero',
     'isnotzero',
     'todict',
     'tocoords',
     'jac',
     'hess',
     'hess3',
     'bilinearform',
    ...
    ```

  


| Home | Trees | Indices | Help | PyDSTool |
| --- | --- | --- | --- | --- |

|  |  |
| --- | --- |
| Generated by Epydoc 3.0.1 on Fri May 4 15:24:03 2012 | http://epydoc.sourceforge.net |
